# Does time of surgery influence the rate of false-negative appendectomies? A retrospective observational study of 274 patients

**DOI:** 10.1186/s13037-018-0180-2

**Published:** 2018-12-14

**Authors:** Kirit Singh, Michael S. J. Wilson, Maria Coats

**Affiliations:** 1 Department of General Surgery, Ninewells Hospital, NHS Tayside, Dundee, UK; 2 Cardiff University, Cardiff, UK

**Keywords:** Negative appendectomy, Out of hours, After hours, Off hours, Diagnostic accuracy

## Abstract

**Background:**

Multiple disciplines have described an “after-hours effect” relating to worsened mortality and morbidity outside regular working hours. This retrospective observational study aimed to evaluate whether diagnostic accuracy of a common surgical condition worsened after regular hours.

**Methods:**

Electronic operative records for all non-infant patients (age > 4 years) operated on at a single centre for presumed acute appendicitis were retrospectively reviewed over a 56-month period (06/17/2012–02/01/2017). The primary outcome measure of unknown diagnosis was compared between those performed in regular hours (08:00–17:00) or off hours (17:01–07:59). Pre-clinical biochemistry and pre-morbid status were recorded to determine case heterogeneity between the two groups, along with secondary outcomes of length of stay and complication rate.

**Results:**

Out of 289 procedures, 274 cases were deemed eligible for inclusion. Of the 133 performed in regular hours, 79% were appendicitis, compared to 74% of the 141 procedures performed off hours. The percentage of patients with an unknown diagnosis was 6% in regular hours compared to 15% off hours (RR 2.48; 95% CI 1.14–5.39). This was accompanied by increased numbers of registrars (residents in training) leading procedures off hours (37% compared to 24% in regular hours). Pre-morbid status, biochemistry, length of stay and post-operative complication rate showed no significant difference.

**Conclusions:**

This retrospective study suggests that the rate of unknown diagnoses for acute appendicitis increases overnight, potentially reflecting increased numbers of unnecessary procedures being performed off hours due to poorer diagnostic accuracy. Reduced levels of staffing, availability of diagnostic modalities and changes to workforce training may explain this, but further prospective work is required. Potential solutions may include protocolizing the management of common acute surgical conditions and making more use of non-resident on call senior colleagues.

## Background

Recently, there has been much debate over the existence of a ‘weekend effect,’ accounting for excess mortality in those patients treated at the weekend [1]. Recent discourse was largely initiated by Freemantle et al. in 2012 who described a 16% increase in 30-day mortality between patients admitted on a Sunday compared to a Wednesday [2]. This has since been challenged, with follow-up work by the same group not explicitly linking day of the week to mortality, but still describing a statistical variation [3]. However, despite much of the current focus on these two studies, this variation has been described for several decades, across many specialties [4–8]. This effect on mortality has been seen both in narrow subsets of patients (e.g. those presenting with myocardial infarctions), and more broadly in all patients admitted at the weekend [9–11]. Surgery is no exception to this and similar results have been reported in both the elective and non-elective surgical caseloads in studies focussing on procedures performed on Saturdays or Sundays [12, 13].

Given this large body of sometimes conflicting evidence, finding explanations for this variation are challenging. Concha et al. suggested that the perceived suboptimal quality of care may be caused by reduced staffing levels or that patients who present outside of regular working hours have a greater burden of comorbidities with a worse pre-morbid state [14]. Bray et al. examined this effect in 74,307 stroke patients finding several patterns of weekly variation, not just at the weekend [15]. They go on to argue that the notion of a weekend effect is likely something of an oversimplification and that future investigatory work should focus on reducing variation in quality [15].

This variation in weekly patterns is again reflected in some surgical studies that have found evidence of poorer morbidity and mortality outcomes from procedures performed overnight. One study examining non-elective laparoscopic cholecystectomies performed in the evening reported an odds ratio as high as 3.33 for post-operative complications [16] although other studies describe no such variation [17]. It is notable however, that studies examining variation in care are often naturally focussed on morbidity and mortality, while there is little evidence regarding the diagnostic accuracy of procedures performed outside of regular working hours. This is despite these procedures being performed in a clinical setting with reduced staffing levels and with limited availability of diagnostic modalities.

Therefore, the primary aim of this retrospective observational analysis was to test the hypothesis that diagnostic accuracy would be reduced in the off hours setting, given the aforementioned challenges. To demonstrate this, management of a common acute surgical presentation was to be reviewed, namely acute appendicitis, with procedures resulting in an unknown pathology deemed to be a false-negative diagnosis. Appendicitis is one of the commonest general surgical presentations, with a lifetime risk of 7–8% and 34,600 cases reported a year in England alone [18, 19], but also can be one of the more challenging to diagnose, with vague symptoms initially and a wide differential. Accordingly, a false-negative appendicectomy rate of anywhere up to 25% has been historically deemed acceptable, with rates even higher in women due to the broader range of possible pathology [20, 21]. Given the difficulty in accurately diagnosing this condition and the large number of presentations, this was deemed to be a sensitive marker for variation in diagnostic accuracy.

Secondary aims included measures to evaluate if there was any concurrent variation in case mix between patients admitted off hours and in regular hours by reviewing pre-morbid status and pre-operative diagnostic tests. Theatre records including length of procedure, ASA status and grade of primary surgeon were also collected. Further, post procedural outcomes including length of stay and complication rates were reviewed.

## Methods

Patients were selected retrospectively from emergency theatre records logged in the Centricity™ Opera database (General Electric Healthcare) in a single centre over 56 months from 17th June 2012 to 1st February 2017, representing the complete set of records available since use of this database commenced. All non-infant patients (i.e. age > 4 years) who presented to the surgical admissions unit within the centre who were subsequently taken for an operation for presumed appendicitis were deemed eligible for inclusion. Cases were excluded if a formal diagnosis was not recorded histologically and/or on the discharge summary document.

Patients were classified according to their final confirmed diagnosis which comprised of three cohorts; appendicitis, other pathology (including irritable bowel disease, neoplasm, gynaecological and other miscellaneous findings) and unknown (no clear diagnosis either histologically or on the discharge record).

Data were also gathered on the grade of primary surgeon (registrar or consultant, equivalent to resident in training or attending surgeon respectively), as well as patient demographics, length of stay and complication rates (within 30 days including readmissions related to their surgery). Pre-operative data was also recorded, including; American Society for Anaesthetists (ASA) status, white cell count (WCC) and c-reactive protein (CRP) to determine the heterogeneity between the cases sampled.

Patients were stratified into two groups: ‘regular hours’ or ‘off hours’ based on the time they arrived in the anaesthetic room. Regular hours were defined as 08:00 to 17:00, reflecting the normal daily duration of consultant cover on the surgical admissions unit (including at Saturday and Sunday) while off hours were defined as 17:01 to 07:59 where staffing numbers fell to on-call levels.

The primary outcome measure of this study used to reflect diagnostic accuracy was the unknown diagnosis rate, with the hypothesis being supported if a greater number of unknown diagnoses occurred in the off hours settings. If patients were taken for emergency surgery querying appendicitis and their histological/discharge diagnosis was unknown, this was classified as a negative diagnosis. Secondary outcomes included post-operative length of stay and complication rate.

Statistical analysis was performed using SPSS (version 25). All recorded parameters were evaluated using descriptive statistics and the relative risk of a negative diagnosis was calculated between the off hours and regular hours patient group with a significance level of 0.05. Ethical approval was sought through the Caldicott Guardian to access patient records. All data was stored on a secured database in a password protected file with patient identifies anonymised.

## Results

Over the 56-month period, 289 patients underwent emergency surgery for presumed appendicitis within the institution. Of these cases, 274 patients were identified for inclusion into the study (59.1% male, *n* = 162: 40.9% female, *n* = 112) with 15 cases excluded due to no confirmed diagnosis being recorded either histologically or on discharge documents. In regular hours, 133 patients underwent emergency surgery. The mean age of this group was 32.6 years (± 2.916) and of these, 78.9% had a confirmed diagnosis of appendicitis. In off hours, 141 patients were operated on, with a mean age of 32.2 (± 3.236) of whom 73.8% were confirmed to have appendicitis (summarised in Table 1).

**Table 1:** Patient Episode Characteristics

Variable	In Hours (*n* = 133)	Out of Hours (*n* = 141)
Mean Age ± SD	32.617 ± 2.916	32.17 ± 3.236
Operating Time, hrs		
Mean ± SD	1.253 ± 0.106	1.17 ± 0.093
Grade of Surgeon, n (%)		
Consultant Lead	101 (76%)	89 (63%)
Registrar Lead	32 (24%)	52 (37%)
Length of Stay		
Mean ± SD	3.86 ± 0.729	3.577 ± 0.749
Complications (%)	18 (13.5%)	17 (12.1%)

**Fig. 1:**
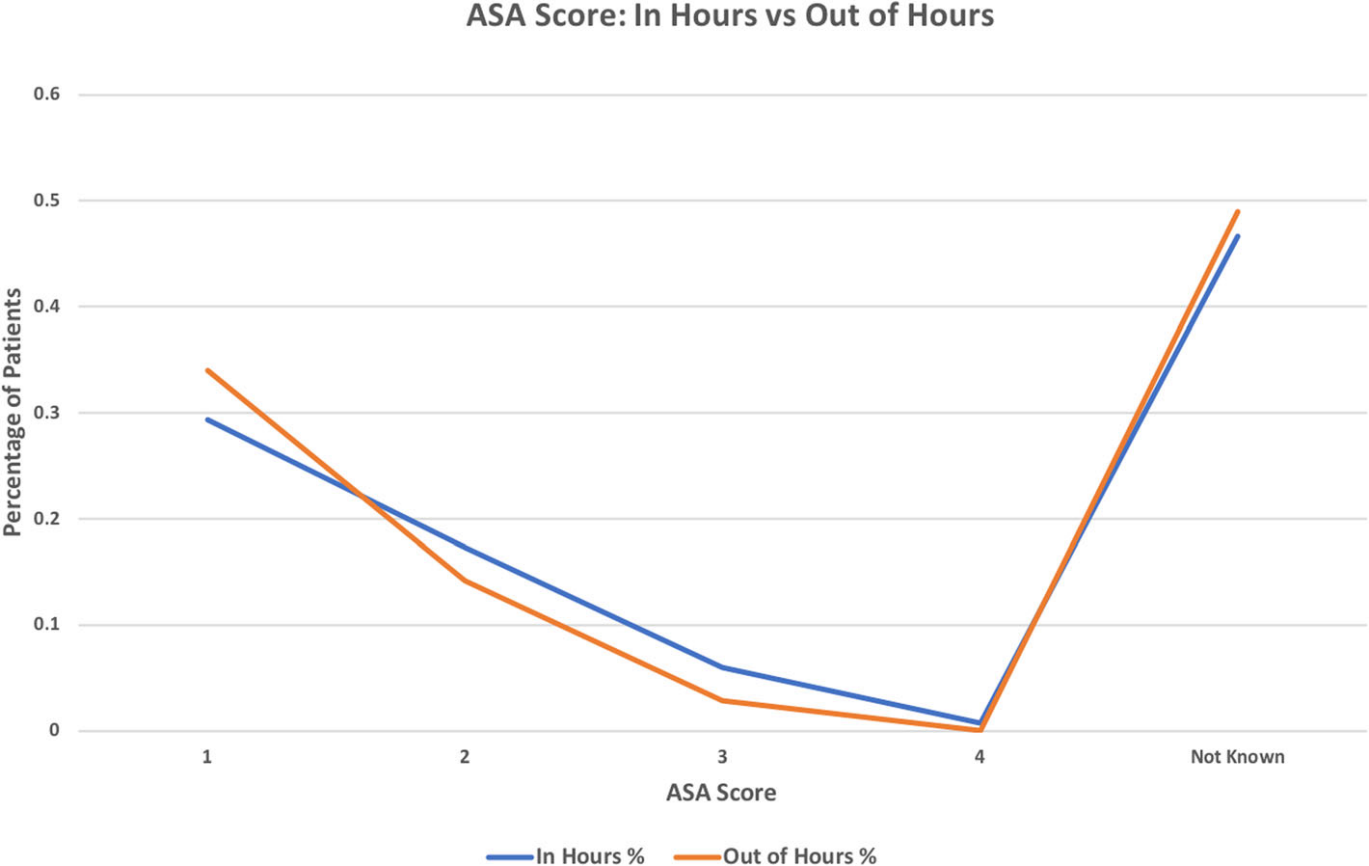
ASA Score: Regular Hours vs Off Hours

Pre-operatively, abnormal biochemistry markers were observed in 86.5% of cases admitted in regular hours, while similarly 85.1% of off hours patients were found to be abnormal. Negligible difference was also observed in pre-operative ASA status (Table 2) between regular and off hours cases (Fig. 1) indicating a high degree of homogeneity between the two groups.

**Table 2:** Patient Pre-Morbid Status

ASA Status	No. of Patients (%)	
	In Hours (*n* = 133)	Out of Hours (*n* = 141)
I	39 (29.3%)	48 (34.0%)
II	23 (17.3%)	20 (14.2%)
III	8 (6.0%)	4 (2.8%)
IV	1 (0.8%)	0 (0.0%)
Not Known	62 (46.6%)	69 (48.9%)

The primary outcome measure of diagnostic outcomes showed no significant variation between regular and off hours for rates of appendicitis or other pathology. However, there was a significant difference between rates of unknown diagnoses, comprising 14.9% of cases operated on off hours compared to 6.0% of cases operated on in regular hours (Table 3).

**Table 3:** Comparison of Diagnostic Outcomes

Diagnosis	No. of Patients (%)	
	In Hours (*n* = 133)	Out of Hours (*n* = 141)
Appendicitis	105 (78.9%)	104 (73.8%)
Other	20 (15.0%)	16 (11.3%)
Unknown	8 (6.0%)	21 (14.9%)

The relative risk of having an unknown diagnosis from a procedure performed off hours was 2.5 times that of one performed in regular hours (RR 2.48; 95% CI 1.14–5.39). The only other difference observed between the two patient groups was the grade of the lead surgeon, which was registrar (or resident in training) led in 24% of cases in regular hours, rising to 37% in the off hours setting.

Secondary outcome measures of length of stay showed no significant difference with a mean of 3.9 days for regular hours patients, compared to 3.6 for off hours patients, while 30-day complication rates were also similar at 13.5 and 12.1% respectively.

## Discussion

Following our review of this 56-month period, the hypothesis that diagnostic accuracy is reduced in the off hours setting would seem to be confirmed with patients having nearly 2.5 times the rate of an unknown diagnosis than those undergoing procedures in regular hours. This finding may therefore reflect an increased number of unnecessary emergency operations being undertaken, exposing patients to increased risks and side effects for no benefit if admitted or operated on outside of regular working hours. No other significant difference between the two groups was found, apart from the grade of lead surgeon recorded, with registrar led procedures increasing by a third (from 24 to 37%) in the off hours setting. However, this does not necessarily confer a causative relationship and the reasons for any such link are likely complex. Pre-operative morbidity (characterised by ASA status) in both groups was nearly exactly the same and no significant variation was found in the length of stay or 30-day post-operative complication rates. However it is notable that in this centre the negative diagnosis rate for both groups (at 6.0% in regular hours and 14.9% for off hours patients) both remain below rates historically considered acceptable [22], with minimal variation of positive diagnoses of appendicitis between the two cohorts.

An initial concern when gathering the data was that although the defined time periods of off hours and regular hours mirrored the working patterns of the hospital. The off hours period, as defined in this study, was twice as long as the regular hours period, potentially skewing the results. However, our data has found that of the 274 records included, 48.5% of procedures were performed in regular hours, while 51.5% were performed off hours, a near even balance. Further adding confidence to the heterogeneity of the case mix analysed was the lack of variability in ASA status and pre-operative biochemistry between the two groups, which is likely to be expected for emergency admissions relating to acute appendicitis.

As mentioned before, while appendicitis is a common surgical presentation, it can also be non-specific in its symptoms and often mimicked by other pathologies. In the off hours setting, with less diagnostic modalities available for borderline cases, the decision to not operate becomes more challenging, especially when balancing intervention against the risk of more serious sequelae such as perforation [23]. Changes made to training pathways resulting in a decrease in working hours and exposure to such unclear cases may also be playing a role in this variation [24]. Decisions on operating off hours are often also made in a higher stress environment as staffing levels fall and individual workload increase. Relative inexperience and a higher workload have been previously recognized as playing a role in worsening of outcomes [25, 26], and it would be unsurprising to also find these also playing a deleterious role in diagnostic decision making.

It is also noteworthy that there appeared to be no significant variation in our study of complication rate or length of stay between the two groups. This aligns with prior research which shows that appendicectomies performed by surgical trainees has similar outcomes to procedures performed by consultant surgeons [27]. Our findings would therefore suggest that any potential variation in quality exists in the pre-procedure stage, rather than intra or post-operatively.

## Solutions

While it is difficult to improve upon staffing levels or diagnostic availability in the off hours setting without increasing healthcare resource allocation, there may be simpler ways to support decision making. The data gathered shows that the vast majority of off hours procedures are initiated within the 17:01–00:00 period (87%) and accordingly that non-resident on call consultants who provide senior cover for advice would likely be readily available to discuss borderline cases. While appendicitis is seen as an index surgical diagnosis, it may be that encouraging discussions with senior surgeons when off hours staff are uncertain could provide added insight and reduce the rate of unknown diagnoses occurring outside of regular hours, due to fewer unnecessary procedures.

A protocolized approach to pre-operative workup may also ensure standardization of the decision over when to operate. While prior research has suggested that history and examination may be sufficient for the majority of patients [28], protocolizing management may provide some benefit for those difficult to assess. Such protocols for diagnosing and managing appendicitis have already been suggested but challenges remain in this regard [29, 30]. Several scoring systems for acute appendicitis (such as MANTRELS or Alvarado) have been found to not meet performance benchmarks, and although it has been suggested that negative biochemical markers of inflammation could be used to rule out acute appendicitis, this remains a topic of some debate [31, 32].

## Limitations

Interpretation of the above findings must also consider several sources of bias that may have occurred. Firstly, retrospective studies relying on a mixture of data may lack reliability. This is especially the case when analysing electronically generated records for diagnoses, which although must be entered on a discharge summary, may often be produced by a ward doctor unfamiliar with the case and therefore erroneous. Error also may arise by relying on theatre logs that have not been validated against paper records detailing the grade of the lead surgeon or the intra-operative findings. This may influence the rate of unknown procedures being performed by registrars, as our study has described an improbably high number of off hours procedures with a consultant listed as the lead surgeon, despite them being non-resident on call in that period. This could well underrepresent the rate of registrars performing procedures resulting in an unknown diagnosis outside of regular working hours.

Records were also allocated to regular or off hours based on emergency theatre logs and the point at which the patient arrived in the anaesthetic room. Ultimately, this is a crude measure and may not accurately reflect the decision-making process. Patients admitted in regular hours may be seen by the consultant leading that day’s patient intake but operated on in the off hours period as clinical necessity dictates. However, this analysis assumes that all cases arriving after 17:00 have had their treatment pathway decided by the off hours covering team, which consists of different doctors to those working the regular day shift. This may be especially problematic for patients who begin their procedure close to the boundaries of the two periods as they may well have been seen on the acute surgical receiving unit several hours earlier and had their management determined by senior colleagues. This crude measure could be refined if data on time of patient presentation was available from retrospective records but unfortunately this was not collected in the databases analysed.

Similarly, using ASA status and pre-operative biochemistry to assume heterogeneity of the cohorts is a crude measure. In addition, the majority of cases had no entry recorded relating to ASA status. While our data found that pre-morbid results correlated closely between the two groups, this large number of missing records ultimately means there is significant room for variation.

Given that our analysis appears to show variation occurring within the pre-procedure setting, future work must focus on this decision-making pathway. This would likely require a prospective design which validates electronic records with paper operative notes, and also documents the lead clinical decision maker as well as the lead surgeon. Furthermore, without a clear documentation of the time at which the decision to operate was made, or recording the initial time of patient presentation, it remains difficult to conclusively determine whether said variation in diagnostic accuracy between off hours and regular hours truly exists.

## Conclusions

The ‘after hours effect’ has thus far largely been attributed to morbidity, mortality and post-operative complications, we now conclude that this may also be extended to the pre-procedure setting, in the form of diagnostic accuracy, with more unknown diagnoses stemming from procedures performed in the off hours setting compared to those under taken in regular working hours. However, despite this variation, our findings demonstrated that the frequency of negative diagnoses in the two cohorts were well below historical rates. Nevertheless, a service working with reduced staffing levels, reduced availability of diagnostic modalities on a backdrop of recent changes to workforce training may explain increased rates of procedures resulting in unknown diagnoses, but further work is required to definitively prove this hypothesis. Potential solutions may include protocolizing the management of common acute surgical conditions to prevent unnecessary procedures being performed in the off hours setting, while also making more use of non-resident on call senior colleagues for difficult or atypical cases.

## Abbreviations

ASA: American Society of Anaesthetists
CRP: C-Reactive Protein
RR: Relative Risk
WCC: White Cell Count
